# TACE/ADAM-17 Phosphorylation by PKC-Epsilon Mediates Premalignant Changes in Tobacco Smoke-Exposed Lung Cells

**DOI:** 10.1371/journal.pone.0017489

**Published:** 2011-03-15

**Authors:** Hassan Lemjabbar-Alaoui, Sukhvinder S. Sidhu, Aklilu Mengistab, Marianne Gallup, Carol Basbaum

**Affiliations:** 1 Thoracic Oncology Laboratory, Department of Surgery, Comprehensive Cancer Center, University of California San Francisco, San Francisco, California, United States of America; 2 Divisions of Pulmonary and Critical Care and Allergy/Immunology, Department of Medicine, University of California San Francisco, San Francisco, California, United States of America; 3 Biomedical Sciences Program, Cardiovascular Research Institute and Department of Anatomy, University of California San Francisco, San Francisco, California, United States of America; Dana-Farber Cancer Institute, United States of America

## Abstract

**Background:**

Tobacco smoke predisposes humans and animals to develop lung tumors, but the molecular events responsible for this are poorly understood. We recently showed that signaling mechanisms triggered by smoke in lung cells could lead to the activation of a growth factor signaling pathway, thereby promoting hyperproliferation of lung epithelial cells. Hyperproliferation is considered a premalignant change in the lung, in that increased rates of DNA synthesis are associated with an increased number of DNA copying errors, events that are exacerbated in the presence of tobacco smoke carcinogens. Despite the existence of DNA repair mechanisms, a small percentage of these errors go unrepaired and can lead to tumorigenic mutations. The results of our previous study showed that an early event following smoke exposure was the generation of oxygen radicals through the activation of NADPH oxidase. Although it was clear that these radicals transduced signals through the epidermal growth factor receptor (EGFR), and that this was mediated by TACE-dependent cleavage of amphiregulin, it remained uncertain how oxygen radicals were able to activate TACE.

**Principal Findings:**

In the present study, we demonstrate for the first time that phosphorylation of TACE at serine/threonine residues by tobacco smoke induces amphiregulin release and EGFR activation. TACE phosphorylation is triggered in smoke-exposed lung cells by the ROS-induced activation of PKC through the action of SRC kinase. Furthermore, we identified PKCε as the PKC isoform involved in smoke-induced TACE activation and hyperproliferation of lung cells.

**Conclusions:**

Our data elucidate new signaling paradigms by which tobacco smoke promotes TACE activation and hyperproliferation of lung cells.

## Introduction

Smoke exposure is the principal risk factor for the development of lung cancer [1], [2], but details of the pathogenesis are unknown. In general, tumor progression occurs in temporally and spatially overlapping stages consisting of premalignant hyperplasia, dysplasia, carcinoma in situ and invasive cancer [3].

We previously reported that hyperplasia of lung cells was elicited by smoke-induced phosphorylation of the epidermal growth factor receptor (EGFR) [4], [5]. This suggested that an understanding of the mechanism by which smoke activates EGFR might elucidate lung cancer pathogenesis and prompted us to identify mechanisms linking EGFR activation to tobacco smoke. In our previous study [4] we identified a cascade of smoke-induced signaling events, the earliest of which was the generation of intracellular reactive oxygen species, ROS. In principle, this could have occurred either by release of ROS from mitochondria or by the generation of ROS de novo by NADPH oxidase. Inhibitor studies showed that the increased ROS levels in smoke-exposed cells were due to activation of NADPH oxidase and we were able to link smoke-induced ROS to EGFR activation by the observation that smoke caused the ROS-dependent release of a soluble EGFR ligand, amphiregulin.

Amphiregulin is one of a family of EGFR ligands (EGF, beta-cellulin, HBEGF, TGFα, etc) that begin life as transmembrane proteins and are eventually cleaved (“shed”) from the cell surface, enabling them to bind to, and thereby activate EGFR [6], [7], [8], [9]. The cleavage event is commonly mediated by one of a family of cell surface metalloproteinases called ADAMs (A Disintegrin and Metalloproteinase) [10], [11]. ADAM metalloproteinases are a branch of the metzincin metalloproteinase superfamily that are related to snake venom metalloproteinases and integrin ligands [11], [12], [13]. They comprise more than 40 cell surface transmembrane proteins whose functions run the gamut from proteolytic processing or "shedding" of cell surface protein ectodomains to cell adhesion, membrane fusion, and intracellular signaling [11], [13]. They contain modular metalloproteinase, disintegrin, and cysteine-rich, epidermal growth factor-like domains, followed in most cases by a transmembrane region and cytoplasmic domain [11], [13]. One of the best characterized is tumor necrosis factor-convertase (TACE, ADAM17) [14]. Our experiments confirmed that a metalloproteinase was required for smoke-induced amphiregulin release and identified the specific metalloproteinase as TACE/ADAM 17 [4].

The signaling pathways controlling ADAM function are presently unclear. Elucidation of the mechanisms that govern TACE activation and cleavage of EGF family shedding is critical for the understanding of the regulation of EGFR. The cytoplasmic tail of TACE contains serine, threonine and tyrosine residues that represent potential phosphorylation sites. The phosphorylation of one or more of these sites has been observed in response to diverse extracellular stimuli [15], [16], [17], [18], but the issue of whether or not there is a functional link between phosphorylation and catalytic activity remains controversial. Intriguingly, Grandis and colleagues have demonstrated that Gastrin releasing peptide (GRP)/GRP receptor autocrine pathway can transactivate EGFR head and neck cancer cell lines. This EGFR transactivation is mediated through GRP-induced TACE phosphorylation (on serine and threonine residues) and amphiregulin release [19]. Another stimulus known to cause protein shedding by ADAMs is TPA, a phorbol ester [20], [21]. TPA is a known tumor promoter, which perturbs intracellular signaling by activating PKC. It has been shown that PKCε activation is an initial signal in TPA-induced TACE activation and shedding of TNFα from epidermal keratinocytes [22]. Taken together, these reports suggest that phosphorylation by PKC may control TACE activity.

Here we show that TACE phosphorylation is induced by tobacco smoke in a PKCε-dependent manner and that this is associated with enhanced TACE activity. In this context, PKC activation is dependent upon ROS and their downstream effector, SRC.

Our data identify a novel mechanism regulating TACE sheddase activity, which may have important implications for the known roles of TACE in the release of EGFR ligands, cell hyperplasia and carcinogenesis.

## Results

### Smoke induces TACE phosphorylation and activation

**Figure 1:**
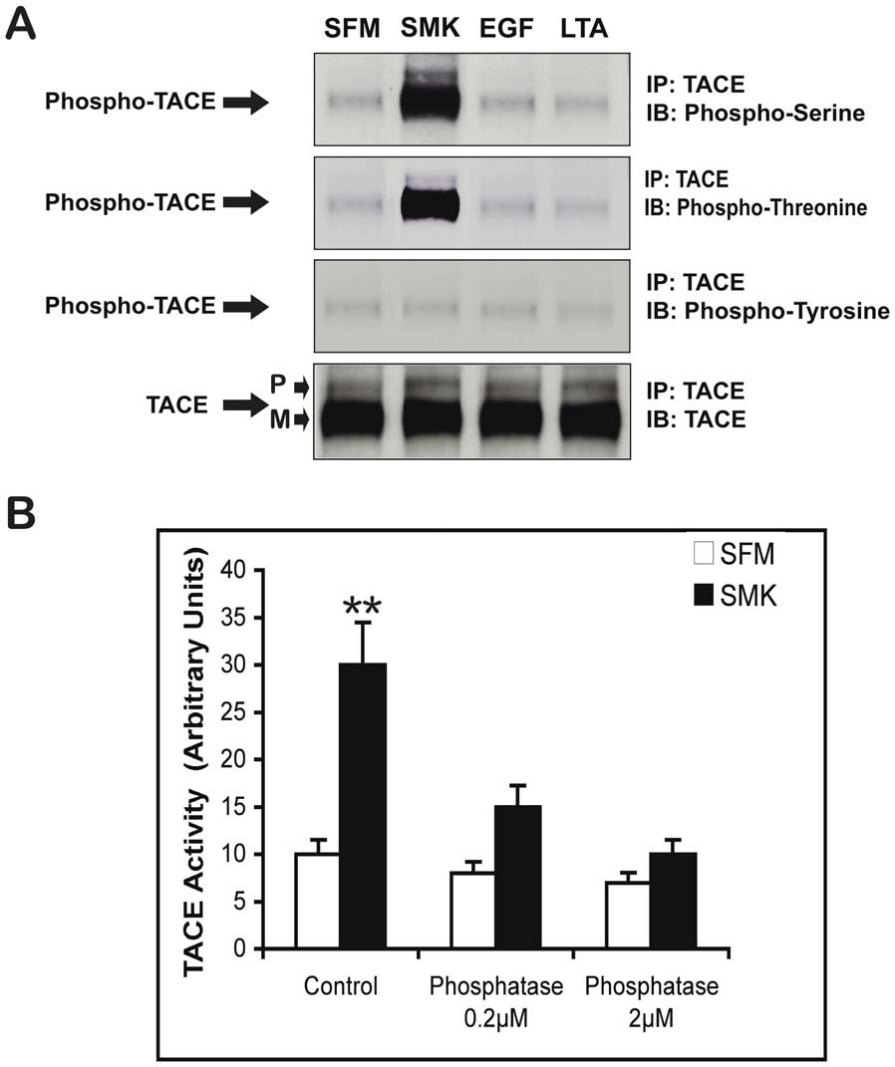
Smoke stimulates phosphorylation and activation of TACE. (A) NCIH292 cells were incubated with SFM, smoke containing SFM (SMK), EGF (10 ng/ml)-containing SFM, or LTA (50 µg/ml)-containing SFM for 10 min. Cell lysates were immunoprecipitated (IP) with anti-TACE antibody, immunoblotted (IB) with anti-phosphoserine, antiphosphothreonine, anti-phosphotyrosine and anti-TACE antibodies, and visualized by chemiluminescence. P and M indicate the Pro- and Mature forms of TACE, respectively. (B) TACE activity was measured using the fluorescent InnoZyme™ TACE activity Kit. NCIH292 cells were incubated with SFM, smoke containing SFM (SMK) for 10 min then harvested. Total cell lysates were prepared and TACE activity was measured according to the manufacturer's recommended protocol. RFU is reported per mg protein. To assess the relevance of phosphorylation on TACE catalytic activity, NCIH292 cells were incubated with phosphatase at the indicate concentrations for 2 h prior to stimulation with smoke (SMK) for 10 min. Double asterisks indicate significantly different from SFM (p<0.01).

As described previously, we exposed lung epithelial (NCI-H292) cells to tobacco smoke condensate for 10 minutes, at which time TACE-dependent amphiregulin cleavage and phosphorylation of EGFR was maximal [4]. Our aim in the first set of experiments was to determine whether TACE activation by smoke was accompanied by TACE phosphorylation. Figure 1A shows that smoke induces TACE phosphorylation on serine and threonine residues, but not tyrosine. TACE phosphorylation was not effected by exogenous EGF or purified LTA from S. aureus which induces cleavage of HBEGF in a ADAM-10 dependent manner in lung cells [23]. We next investigated the possibility that phosphorylation affected TACE function. Figure 1B shows that the activity of TACE, immunoprecipitated from smoke-exposed cells, increased by 3-fold compared to TACE immunoprecipitated from non-smoke-exposed cells. Moreover, when immunoprecipitated TACE was dephosphorylated (by alkaline phosphatase) prior to assay, it lost much of its activity. Thus, tobacco smoke elicits TACE phosphorylation, which is required for smoke-induced TACE activity against a synthetic substrate.

### Smoke exposure activates PKC

**Figure 2:**
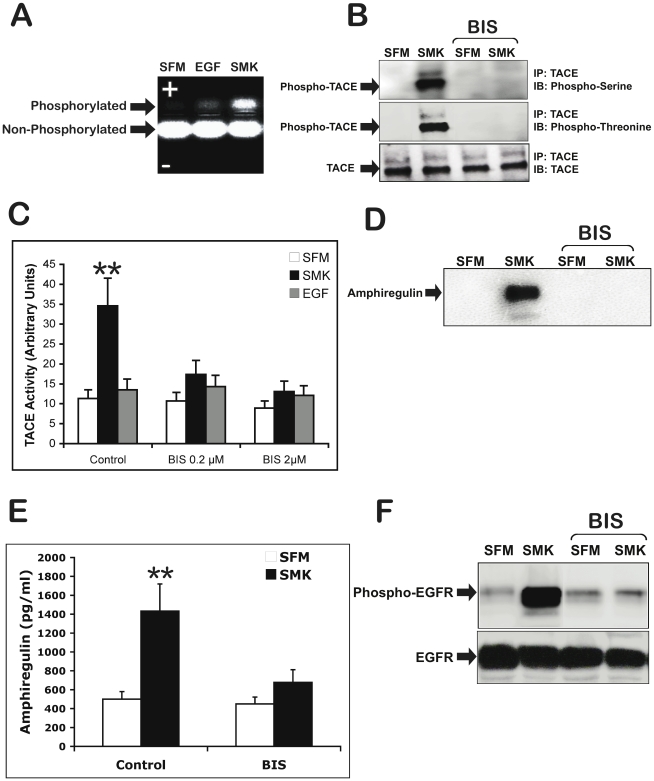
Protein Kinase C (PKC) is required for Smoke-induced TACE activation. (A) PKC activity was detected by using PepTag(®) assay, a non-radioactive detection method of Protein Kinase C. NCIH292 cells were incubated with SFM, EGF (10 ng/ml)-containing SFM, or smoke containing SFM (SMK) for 10 min then harvested. Cell extracts samples for PKC assay were prepared following the protocol recommended by the manufacturer. Detection of PKC activity in cell extract was carried out according to the manufacturer's recommended protocol. (+) indicates anode and (−) indicates cathode. (B) NCIH292 cells were incubated with 5 µM of the general PKC inhibitor, bisindolylmaleimide (BIS), for 2 h prior to stimulation with smoke (SMK) for 10 min. Cell lysates were immunoprecipitated (IP) with anti-TACE antibody, immunoblotted (IB) with anti-phosphoserine, antiphosphothreonine, and anti-TACE antibodies, and visualized by chemiluminescence. (C) To assess the relevance of PKC activity on TACE catalytic activity, NCIH292 cells were incubated with BIS at the indicate concentrations for 2 h prior to stimulation with smoke (SMK) for 10 min. Total cell lysates were prepared and TACE activity was measured as described in Fig. 1. Double asterisks indicate significantly different from SFM (p<0.01). (D) Following incubation with SFM, smoke containing SFM (SMK), cell culture medium was collected and incubated with heparin-Sepharose to precipitate EGFR ligands. Immunoblot was carried out with amphiregulin specific antibody. (E) An amphiregulin ELISA was performed on cell culture media according to the manufacturer's instructions. Cumulative results are shown from 3 independent experiments. (F) To assess the relevance of PKC activity on SMK-induced EGFR activation, cell lysates were immunoprecipitated (IP) with anti-EGR antibody, immunoblotted (IB) with anti-phosphotyrosine, and anti-EGFR antibodies, and visualized by chemiluminescence.

How does smoke induce TACE phosphorylation? Clues can be found in evidence showing that TPA is a potent activator of TACE [21], [24] and that TPA exerts its major effects via the activation of protein kinase C (PKC) [25]. This led us to ask whether PKC might be part of the signaling cascade initiated by smoke. As shown in Figure 2A, PKC activity was indeed stimulated by smoke exposure. Moreover, the PKC inhibitor Bisindolylmaleimide (BIS) abrogated smoke-induced TACE phosphorylation (Fig. 2B) and inhibited smoke induced TACE activity (Fig. 2C). In addition, smoke failed to induce amphiregulin release into the medium in the presence of BIS (Figs. 2D–E). Thus, activation of PKC is required for smoke-induced TACE activation and amphiregulin shedding. In keeping with this data, PKC inhibitor BIS also blocked smoke-induced phosphorylation of EGFR (Fig. 2F). Of note, Vehicle control for BIS treatment (i.e., medium containing 0.1% DMSO) was without effect on smoke-induced TACE phosphorylation/activation, EGFR phosphorylation, PKC activity or amphiregulin release (Fig. S1).

### ROS are required for PKC activation

**Figure 3:**
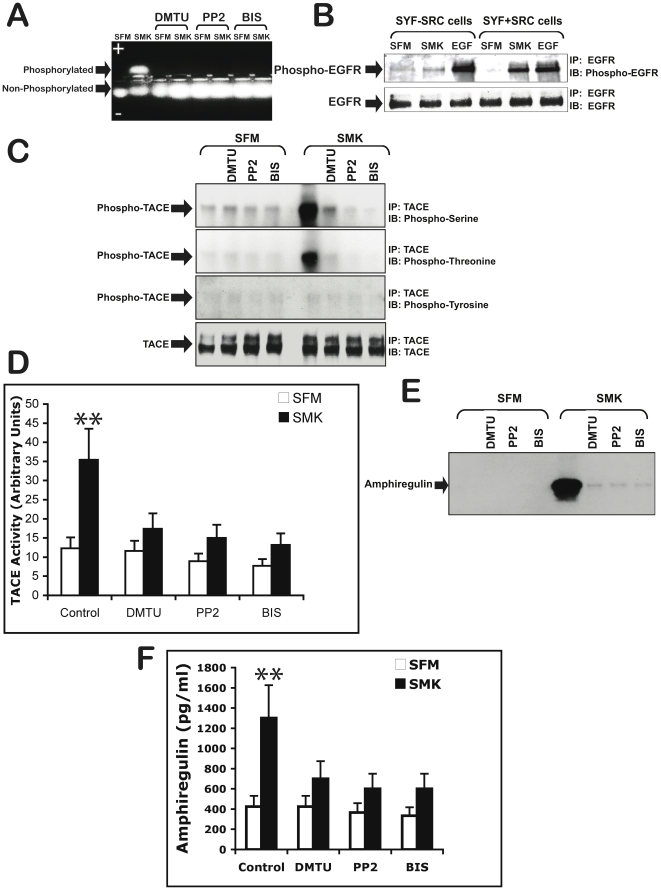
Reactive Oxygen Species (ROS) and SRC kinase are required for both Smoke-induced Protein Kinase C (PKC) activation and TACE activation. (A) NCIH292 cells were incubated for 2 h with oxygen radical scavenger dimethylthiourea (DMTU) (50 µM), or SRC kinase inhibitor (PP2) (10 µM) or PKC inhibitor (BIS) (5 µM), prior to stimulation with smoke (SMK) for 10 min. Cell extracts samples were assessed for PKC activity as described in Fig. 2. (B) SYF cells and SYF+ SRC cells were incubated with SFM, smoke containing SFM (SMK), or EGF (10 ng/ml)-containing SFM for 10 min. Cell lysates were immunoprecipitated (IP) with anti-EGR antibody, immunoblotted (IB) with anti-phosphotyrosine, and anti-EGFR antibodies, and visualized by chemiluminescence. (C) NCIH292 cells were incubated for 2 h with antioxidants dimethylthiourea (DMTU) (50 µM), or SRC kinase inhibitor (PP2) (10 µM) or PKC inhibitor (BIS) (5 µM), prior to stimulation with smoke (SMK) for 10 min. Cell extracts samples were assessed for TACE phosphorylation and (D) TACE catalytic activity as described in Fig. 2. Cell culture medium was collected from the latter cells and assayed for amphiregulin release by (E) immunoblotting assay and (F) ELISA assay as described in Fig. 2.

The PKC superfamily includes six isoforms with regulatory and catalytic domains that can be activated by 1,2-diacylglycerol (DAG) produced from receptor-mediated hydrolysis of inositol phospholipids [26]. However, it has been shown that lipid hydrolysis and DAG are dispensable for the activation of several PKC isoforms [27]. These could be activated, instead, by H2O2-induced tyrosine phosphorylation [26]. Our data in Figure 3A shows that smoke-induced PKC activation was inhibited by ROS scavenger dimethylthiourea (DMTU), suggesting that it may be under regulation from ROS. Consistent with the latter observation, exogenous ROS also stimulated PKC activity in NCIH292 cells (Fig. S2).

### SRC is required in smoke activation of PKC, TACE and EGFR

The results above suggest that PKC is positioned between ROS and TACE in the smoke-triggered signaling network, but leaves open two important questions. First, how do ROS actually stimulate PKC and secondly, how does PKC stimulate TACE? It has been shown that H2O2 can stimulate tyrosine phosphorylation (activation) of PKC via the action of SRC family kinases [28]. In light of our observation that smoke can activate SRC kinase in an ROS-dependent manner [5] we wondered whether PKC might be activated by smoke-induced ROS via SRC. Supporting this hypothesis, we show that the presence of SRC inhibitor PP2 blocked activation of PKC by smoke (Fig. 3A). Consistent with this was the ability of the PKC inhibitor to block smoke-induced phosphorylation of EGFR (Fig. S1) and the ability of SRC inhibitor PP2 to block smoke-induced PKC activation (Fig. 3A). Furthermore, mouse embryonic fibroblasts (MEFs) null for the three SRC family members SRC, Yes and Fyn, (SYF cells) did not phosphorylate EGFR in response to smoke. In contrast, SYF cells stably transfected with c-SRC showed a robust response (Fig. 3B). Taken together, the results shown in Figure 3 implicate SRC in the signaling pathway linking smoke to the phosphorylation of EGFR through PKC. The functional importance of PKC, ROS and SRC to smoke- induced TACE phosphorylation/activation is evident from the ability of their respective inhibitors to block this smoke response (Fig. 3C–D). Additionally, these inhibitors abrogated smoke- induced release of amphiregulin into the medium (Figs. 3E–F).

**Figure 4:**
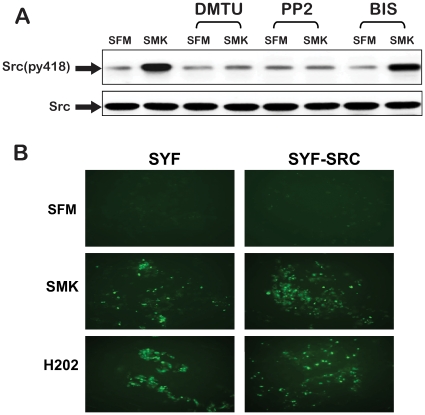
Activation of SRC kinase by smoke is dependent on ROS. (A) NCIH292 cells were incubated for 2 h with oxygen radical scavenger dimethylthiourea (DMTU) (50 µM), or SRC kinase inhibitor (PP2) (10 µM) or PKC inhibitor (BIS) (5 µM), prior to stimulation with smoke (SMK) for 10 min. Cell extracts samples were immunoprecipitated (IP) with anti-SRC antibody, immunoblotted (IB) with anti-phosphotyrosine-418 SRC(py418) reflecting SRC activation, and anti-SRC antibodies, and visualized by chemiluminescence. (B) SYF cells and SYF+SRC cells were loaded with 10 µM H2DCFDA for 30 min prior to stimulation with SFM, smoke containing SFM (SMK), or H2O2-containing SFM (H2O2) for 10 min. Fluorescence indicates the presence of ROS.

Next, we sought to further elucidate the signaling hierarchy linking ROS, SRC and PKC in the smoke-induced PKC activation. We observed that SRC phosphorylation on tyrosine-418 (a reflection of SRC activation) in response to smoke exposure, was inhibited in the presence of the oxygen radical scavenger DMTU as well as in the presence of the SRC inhibitor PP2 but not by the PKC inhibitor BIS (Fig. 4A). This data further supports that SRC is positioned between ROS and PKC in a smoke-triggered signaling network. In line with these observations that smoke-induced ROS generation is SRC independent, we also show that smoke exposure raised the levels of ROS in the MEFs cells null for the three SRC family members (SYF cells) (Fig. 4B). In accordance with these results, exogenous ROS-induced PKC activation was also abrogated in the presence of the SRC inhibitor PP2 (Fig. S2).

### PKCε isoform is required for smoke-induced activation of TACE and EGFR

**Figure 5:**
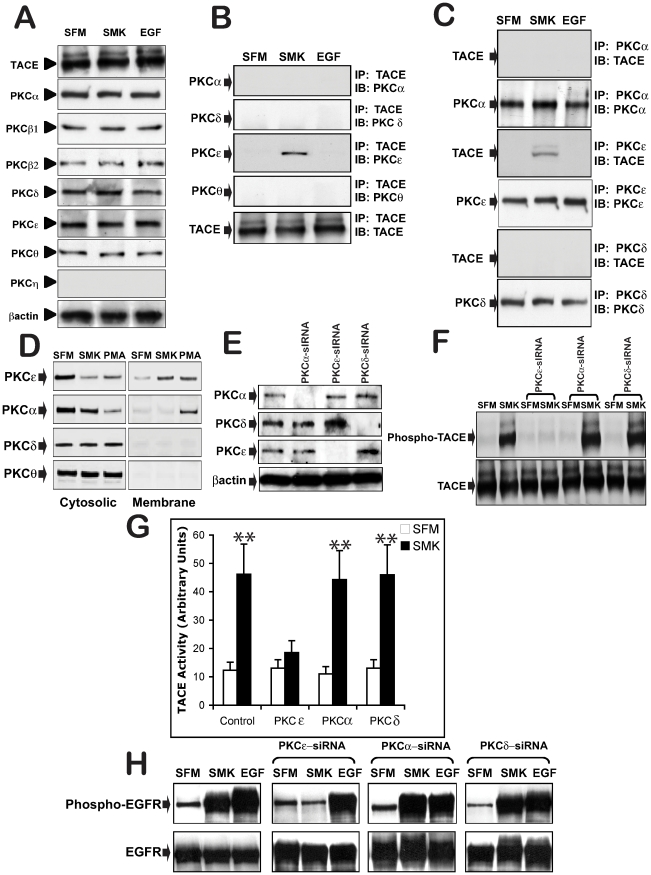
PKCε mediates SMK-induced TACE and EGFR phosphorylation. Smoke stimulates phosphorylation and activation of TACE. (A) NCIH292 cells were incubated with SFM, smoke containing SFM (SMK), or EGF (10 ng/ml)-containing SFM for 10 min. (A) Cell lysates were separated by electrophoresis and immunoblotted with anti-TACE or anti-isoformspecific PKC antibodies as indicated. Actin was used as protein level loading control. (B) Cell extracts samples were immunoprecipitated (IP) with anti-TACE antibody, and immunoblotted (IB) with anti-isoformspecific PKC or anti-TACE antibodies as indicated. (C) Cell extracts samples were immunoprecipitated (IP) with anti-isoformspecific PKC, and immunoblotted (IB) with anti-TACE or anti-isoformspecific PKC antibodies as indicated. (D) Cultured NCIH292 cells were treated for 10 min with SFM, SMK or PMA (400 nM). Cell lysates were fractionated into a membrane and a cytosolic fractions then separated by electrophoresis and immunoblotted with anti-isoformspecific PKC antibodies as indicated. (E) NCIH292 cells were transfected with isoformspecific PKC siRNAs as indicated prior to stimulation with smoke-containing SFM, and immunoblotted with anti-isoformspecific PKC antibodies. Actin is immunoblotted as a loading control. NCIH292 cells or NCIH292 knockdown for PKCε or PKCα or PKCδ were incubated with SFM, smoke containing SFM (SMK), or EGF (10 ng/ml)-containing SFM for 10 min. Cell extracts samples were either (F) immunoprecipitated (IP) with anti-TACE antibody, and immunoblotted (IB) with anti-phosphoserine or anti-TACE antibodies, or (G) prepared and TACE activity was measured as described in Fig. 1. (Double asterisks indicate significantly different from SFM (p<0.01)), or (H) immunoprecipitated (IP) with anti-EGFR antibody, and immunoblotted (IB) with anti-phosphotyrosine or anti-EGFR antibody, antibodies as indicated.

The PKC family of proteins includes 6 isoforms with both a regulatory and catalytic domain (α, β{1 and 2}, δ, ε, θ, and η) [29]. Each isoform, when stimulated, transiently binds substrate. We examined the protein expression pattern of these PKC isoforms in NCIH292 lung cancer cells and were able to confirm expression of PKCα, β1, β2, δ, ε, and θ, but not PKCη (Fig. 5A). We also show that 10 min exposure to smoke or recombinant EGF had no effect on the protein expression level of the PKC isoforms or TACE (Fig. 5A). To test whether TACE and these PKC isoforms can associate either constitutively or upon smoke exposure, we stimulated NCIH292 cells with either medium or smoke followed by co-immunoprecipitation and immunoblotting with antibodies directed against either TACE or each of the six PKC isoforms (α, β1, β2, δ, ε, and θ). As shown in Figure 5B, smoke exposure induced TACE and PKCε association, Where only a PKCε specific antibody stained a protein band in the immunoprecipitated TACE derived from smoke exposed cells and only PKCε immunoprecipitates, derived from smoke exposed cells, stained for TACE (Fig. 5C).

Many PKC isoforms including PKCε are mainly localized in the cytosolic fraction of unstimulated cells and undergo translocation to the cell membranes in activated cells [30]. Treatment of cultured NCIH292 with smoke for 10 min resulted in increased membrane associated PKCε, accompanied with a decreased cytosolic concentration of PKCε. However, smoke was without effect on the subcellular localization of PKCα or PKCδ (Fig. 5D). As positive control, cells were stimulated for 10 min with phorbol 12-myristate 13-acetate (PMA) [31]. PMA induced changes in the subcellular localization of PKCε and PKCα but was without effect on the subcellular localization of PKCδ and PKCθ (Fig. 5D). These results strongly suggest that PKCε is the PKC isoform required for smoke-induced activation of TACE. More convincingly, PKCε, but not PKCδ or PKCα knockdown cells failed to show phosphorylation of TACE following smoke exposure (Figs. 5E–F). This decrease in TACE phosphorylation also coincided with a drastic reduction in TACE activity in smoke induced PKCε knockdown cells (Fig. 5G). Furthermore, PKCε knockdown inhibited smoke-induced EGFR phosphorylation (Fig. 5H). PKCδ or PKCα knockdown had no effect on smoke-induced TACE activity or EGFR phosphorylation (Figs. 5G–H).

### PKCε knockdown suppressed smoke-induced cell growth

**Figure 6:**
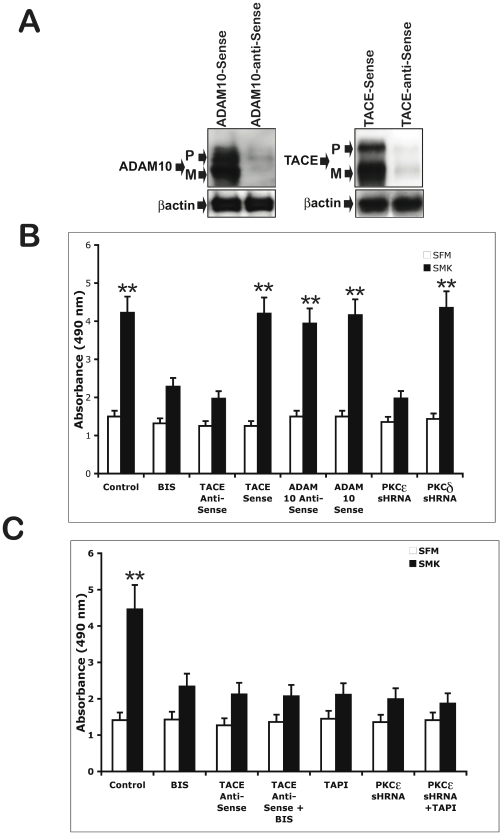
Smoke stimulates PKCε and TACE-dependent proliferation in NCIH292 cells. NCIH292 cells were transfected with 2 µM solutions of morpholino antisense oligonucleotides as indicated prior to stimulation with smoke-containing SFM. (A) Cell lysates were immunobloted with anti-TACE or anti-ADAM10 antibodies. Actin was immunoblotted as a loading control; p indicates pro-enzyme and m indicates mature enzyme. (B) NCIH292 cells (Control) or NCIH292 knockdown for TACE or ADAM10 or PKCε or PKCα or PKCδ were incubated with SFM, smoke containing SFM (SMK) for 10 min. Control cells were incubated for 2 h with PKC inhibitor (BIS) (5 µM), prior to stimulation with smoke (SMK). All cells were assayed for cell growth, using cell Titer assay, 48 h after incubation with SFM or smoke-containing SFM. (C) NCIH292 cells (Control) or NCIH292 knockdown for TACE or PKCε were incubated for 2 h with PKC inhibitor (BIS) (5 µM), or TACE and metalloprotease inhibitor (TAPI) (10 µM) as indicated prior, to stimulation with smoke-containing SFM. Cells were assayed for cell growth, using cell Titer assay, 48 h after incubation with SFM or smoke-containing SFM. Each point in (B) and (C) represents the mean ± S.D of 6 replicates. Double asterisks indicate significantly different from SFM (p<0.01).

We have previously shown that EGFR phosphorylation mediates smoke-induced lung cell hyperplasia in a TACE dependent manner [4], we therefore sought to investigate the role of PKCε in this response to smoke exposure. We employed a previously validated morpholino-antisense siRNA strategy [4] to achieve knockdown of TACE or ADAM10 in H292 cell lines (Fig. 6A). We found that the broad range PKC inhibitor, BIS reduced smoke-induced cell hyperproliferation in lung cells to a level comparable to that induced by TACE knockdown (Fig. 6B). ADAM-10 knockdown was without effect on this smoke response (Fig. 6B). Consistent with our previous data PKCε knockdown inhibited smoke- induced cell hyperproliferation in lung cells whereas no difference in smoke- induced proliferation was observed between PKCα or PKCδ knockdown cells and control cells (mock shRNA knockdown or parental non transduced cells) (Fig. 6B). These findings demonstrate that PKCε promotes cell growth in smoke- exposed lung cells.

Importantly, the addition of the PKC general inhibitor (BIS) to the culture medium of TACE knockdown H292 cells produced no additive inhibition of smoke- induced cell growth (Fig. 6C), consistent with a model in which PKC directly regulates activation. In agreement with these results, pretreatment of PKCε knockdown cells with the MMP and TACE inhibitor (TAPI) produced no further inhibition of smoke- induced cell growth (Fig. 6C). Conversely, TAPI inhibited smoke-induced cell growth in PKCα and PKCδ knockdown cells (data not shown).

### Smoke exposure induces TACE phosphorylation in a PKC-dependent manner in human primary bronchial epithelial cells

**Figure 7:**
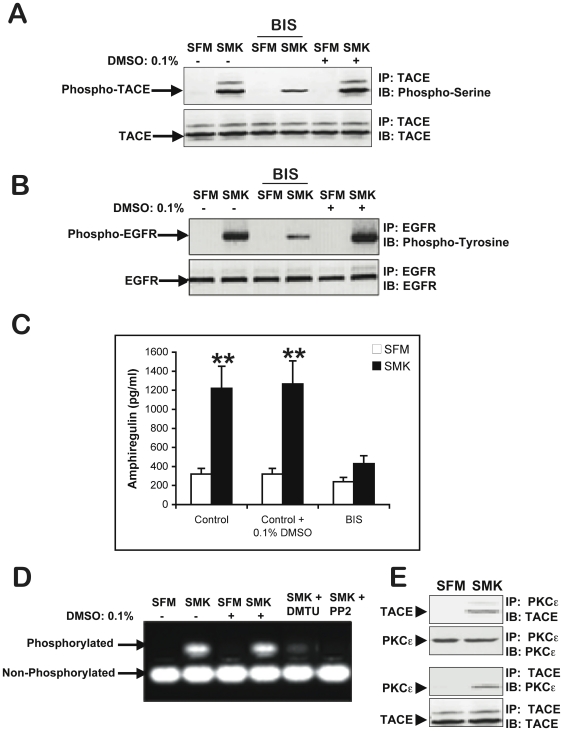
TACE and EGFR activation by smoke in normal primary human bronchial epithelial cells (HBE) is dependent on PKC. Differentiated, pseudostratified HBE cells cultured under air-liquid interface (ALI) were incubated for 2 h with PKC inhibitor (BIS) (5 µM) or medium containing vehicle control (0.1% DMSO), prior to stimulation with SFM or smoke containing SFM (SMK) for 10 min. Cell extracts samples were assessed for (A) TACE phosphorylation and (B) EGFR phosphorylation as described in Figs. 2 and 3. (C) Cell culture medium was collected from the latter cells and assayed for amphiregulin release by ELISA assay as described in Fig. 2. Double asterisks indicate significantly different from SFM (p<0.01). (D) HBE cells in air-liquid interface cultures were incubated for 2 h with oxygen radical scavenger dimethylthiourea (DMTU) (50 µM), or SRC kinase inhibitor (PP2) (10 µM), or medium containing vehicle control (0.1% DMSO) prior to stimulation with smoke (SMK) for 10 min. Cell extracts samples were assessed for PKC activity as described in Fig. 2. (E) HBE cells in air-liquid interface cultures were incubated with SFM or smoke containing SFM (SMK) for 10 min. Cell extracts samples were immunoprecipitated (IP) with anti-TACE antibody or anti-PKCε and immunoblotted (IB) with anti-PKCε or anti-TACE antibodies as indicated.

Since H292 are carcinoma cell line, we then asked whether the changes observed in H292 cells following 10 minutes exposure to smoke extract could be reproduced in differentiated normal primary human bronchial epithelial cells (HBE). As shown in Figure 7A, TACE phosphorylation was indeed stimulated by 10 min smoke exposure of differentiated HBE cells in air-liquid interface cultures. Moreover, smoke ability to stimulate TACE phosphorylation in HBE cells was also inhibited by the PKC inhibitor (BIS) (Fig. 7A). In addition, 10 min smoke exposure also induced EGFR phosphorylation which was blocked by PKC inhibitor Bis (Fig. 7B). Furthermore, smoke induced amphiregulin release into the medium of cultured HBE cells. This Smoke-induced amphiregulin shedding was also abrogated in the presence of Bis (Fig. 7C). Therefore, activation of PKC is also required for smoke-induced TACE and EGFR activation as well as amphiregulin shedding in HBE cells. In keeping, 10 min smoke exposure stimulated PKC activity in HBE cells (Fig. 7D). In agreement with the observations shown above with H292 cells, the smoke-induced PKC activity in HBE cells was also inhibited in the presence of the oxygen radical scavenger DMTU as well as in the presence of the SRC inhibitor PP2 (Fig. 7C). Finally, as observed with H292 cells, 10 min smoke exposure also induced TACE and PKCε association in HBE cells (Fig. 7E). Taken together, these data show that in primary human bronchial epithelial cells, tobacco smoke exposure also elicits TACE activation in a PKC dependent manner.

## Discussion

Previously, we reported that hyperproliferation of lung cells occurs in response to smoke exposure and that this hyperplasia of lung cells is elicited by smoke-induced activation of EGFR [4]. We identified cytoplasmic signaling events induced by tobacco smoke in lung epithelial cells that included oxygen radical-induced cleavage of amphiregulin by TACE and transactivation of EGFR. Smoke-induced binding of amphiregulin to EGFR stimulated epithelial cell proliferation and changes in gene expression within a period of minutes to hours. In addition, previous reports have confirmed that TACE-mediated transactivation of EGF receptors by amphiregulin is important for tumor cell growth and migration [32]. However, to date, the mechanisms underlying the activation of ADAMs are unknown. Understanding these mechanisms are not only important for a basic understanding of “sheddases”, but could also explain smoke-induced TACE activation in the early stages of lung cancer.

The regulation of ADAMs function is complex, involving intracellular proteolytic maturation as well as trafficking to and from the cell membrane [8], [10]. Recent reports have speculated that the cytoplasmic domains of ADAMs might be involved in regulating function or localization to specific sub-cellular structures. Although there is great sequence variability between the cytoplasmic domains of different ADAMs [14], sequences for specific family members are similar across species, arguing that they have important conserved functions. The cytoplasmic domain of many ADAM contains serine, threonine and tyrosine residues, which could be substrates for cytoplasmic kinases. Several studies have suggested that phosphorylation of ADAM cytoplasmic tails may (directly or indirectly) modulate the ability of ADAMs to cleave physiological substrates [16], [17], [19], [33].

In the present study, we demonstrate for the first time that smoke induces TACE phosphorylation at serine/threonine residues in its cytoplasmic domain, and that TACE phosphorylation is required for TACE to cleave EGFR ligands and induce cell hyperproliferation. Phosphorylation of the cytoplasmic tail may induce a conformational change resulting in the exposure of the catalytic zinc ion and the active site pocket making them accessible for substrate binding and thus regulating catalytic activity.

Taking into consideration that the cytoplasmic tail of ADAMs contains PxxP motifs that are binding sites for SH3 domain-containing proteins [34], it is also possible that structural changes induced by phosphorylation, could also lead to changes in the presentation of SH3-binding domains and TACE interaction with SH3 domain-containing proteins. Such interactions have been previously suggested to affect maturation of ADAMs and their localization to a specific membrane domain at the cell surface [35]. Moreover, a phosphorylated cytoplasmic tail could also impact the association of TACE with cytoplasmic proteins by forming SH2 attachment sites for SH2 domain-containing proteins [18]. Consequently, TACE may serve adaptor functions to assemble complexes of proteins at critical sites of functional activity. However, this appears not to be a general mechanism, because the cytoplasmic tail of TACE is dispensable for stimulated shedding of other substrates, such as TNF-α [36], [37]. Although, it is possible that the deletion of the TACE cytoplasmic domain and overexpression of this mutated form may result in its mislocalization. Mislocalized TACE may act in the processing of pro-amphiregulin.

Furthermore, our data show that TACE phosphorylation triggered in smoke-exposed cells is ROS-dependent. This is in agreement with our earlier work demonstrating that ROS production was an early event in smoke-exposed cells and that ROS were required for smoke's TACE-dependent ability to stimulate amphiregulin shedding and thereby phosphorylate EGFR [4]. Although it has been shown that ROS can activate TACE directly via oxidation of a cysteine sulfhydryl group in the prodomain, [24] such a mechanism would not be expected to affect the function of TACE at the cell surface, whose prodomain would have been removed by furin in the Golgi apparatus prior to membrane translocation. Instead, our data shows that ROS are required for protein kinase C (PKC) activation by smoke. This ROS-induced activation of PKC is required for TACE-dependent amphiregulin shedding. It has been speculated that PKC helps to recruit ADAMs to specific sites on the plasma membrane, and upon phosphorylation/activation of the ADAMs, EGFR ligands shedding occurs. In addition we show that smoke-induced PKC activation is mediated by SRC kinase, a finding consistent with a recent report linking H2O2 with SRC kinase [38]. Moreover, another study has shown that the tyrosine phosphorylation of PKC by ROS is mediated by SRC kinase [26], [39], [40]. One possible scenario by which SRC kinase is activated is by the activation of Protein tyrosine phosphatases (PTP) as they carry, highly reactive cysteine residues in their catalytic domains and are sensitive to ROS [41]. Inactivation of PTP leads to elevated levels of protein tyrosine phosphorylation on cellular proteins such as SRC kinases and their subsequent activation. Activated SRC kinase could phosphorylate/activate PKC kinase which in turn activates TACE protein. However, a most recent report revealed that mitochondrial ROS regulate ATP-induced TACE activation and TGFα shedding in CHO cells [42]. In contrast to our findings, this ATP-induced TGF-α shedding was independent of the cytoplasmic NADPH oxidase complex, Src, PKC, and MAPK signaling.

Our present findings provide evidence that the PKC isoform involved in TACE activation is PKCε and that this regulation requires a direct interaction between PKCε and TACE. In agreement with this, TACE activation is suppressed by the selective inhibition of PKCε, but not of PKCδ or PKα. One possible role for PKCε in the triggering of the TACE activation and the shedding of EGFR-ligand is the recruitment of TACE to pro-amphiregulin. If these proteins localize at different membrane domains under steady-state conditions, the movement of TACE to a site that allows interaction with other proteases or with pro-amphiregulin itself might be essential for shedding. The binding and the subsequent phosphorylation of TACE by PKCε may trigger this spatial change in TACE.

**Figure 8:**
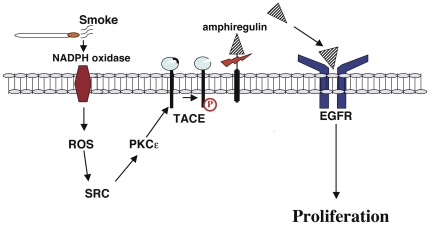
Model for the tobacco smoke-induced transactivation of EGFR via TACE activation. This model combines our earlier results on Smoke-induced signaling in epithelial cells [4] with results from this study. EGFR undergoes phosphorylation in response to tobacco smoke. TACE phosphorylation/activation is triggered in smoke-exposed lung cells by the ROS-induced activation of PKCε through the action of SRC kinase. Smoke –induced TACE activation leads to amphiregulin release, EGFR activation and cell hyperproliferation.

The results of the present study, taken together with our previous results [4] (Fig. 8), provide a missing link into the understanding of how smoke stimulates TACE, EGFR and hyperplasia in lung cells. By analogy with previous work showing that PKCε overexpression and activation by PMA mediates TACE induced-EGFR ligand release and promotes development of skin tumor in mice [22] and also based on the fact that enhanced PKCε activation and expression are required for non small cell lung cancer survival [43], [44], it is conceivable that the stimulation of PKCε by ROS in the smoke-exposed lung may be a major factor in the step of predisposing smokers to develop lung cancer.

## Materials and Methods

### Materials

NCI-H292 cells (mucoepidermoid lung carcinoma), SV40 large T antigen Immortalized embryonic mouse fibroblasts SYF (deficient for SRC, Yes, and Fyn) cells, and SYF+SRC (SFY cells reintroduced with c-SRC) were obtained from American Type Culture Collection (ATCC) (Manassas, VA). All Cell lines were cultured under conditions recommended by the vendor. All tissue culture media and antibiotics were obtained from Invitrogen (Carlsbad, CA) or the University of California, San Francisco, cell culture facility. General PKC (classical and novel) inhibitor bisindolylmaleimide (BIS), SRC kinase inhibitor (PP2) were purchased from Calbiochem (San Diego, CA). Reactive oxygen species (ROS) inhibitor N.N'-dimethylthiourea (DMTU) was purchased from Sigma (St. Louis, MO). MMPs and TACE inhibitor (TAPI-1) was purchased from Santa Cruz Biotechnology (Santa Cruz, CA). Antibodies to EGFR ligands were purchased from R&D Systems, Inc. (Minneapolis, MN). Validated siRNAs to human PKCε (Cat # sc-36251) [45], human PKCα (Cat# sc-36243) [46], and human PKC δ (Cat# sc-36253) [47] were purchased from Santa Cruz Biotechnology (Santa Cruz, CA). 2,7 Dichlorodihydrofluorescein diacetate, H2DCFDA, a cell-permeable substrate for fluorimetric detection of ROS was purchased from Molecular Probes (Eugene, OR). Antibody directed against the cytoplasmic domain of tumor necrosis factor a-converting enzyme (TACE) was from Imgenex (San Diego, CA). All other antibodies were purchased from Santa Cruz Biotechnology (Santa Cruz, CA). LipofectAMINE was purchased from Invitrogen. Purified lipoteichoic acid (LTA) from Staphylococcus aureus and all other reagents (Unless indicated otherwise) were purchased from Sigma.

### Smoke Exposures

Smoke particulates were generated in specially designed animal exposure chambers operated by Dr. Kent Pinkerton at the University of California, Davis [48]. Pall Gelman Pallflex® borosilicate filters (Fisher) were inserted in line during the operation of the chambers. The total suspended smoke particles deposited on each filter were calculated by weighing filters before and after smoke exposure. Filters were mailed to our laboratory at the University of California, San Francisco, where they were stored at 4°C until use. Prior to each experiment, we incubated filters in a volume of SFM to provide a final concentration of 1.0 mg of smoke particulate per ml of SFM. The filters were rotated in this medium at 4°C for 3 days prior to use.

### Immunoprecipitation and Immunoblotting

Immunoprecipitation assays were performed as described previously [4], [23]. Briefly, following treatment with serum free medium containing cigarette smoke extract (SMK), LTA, EGF or control serum free medium (SFM), cells were lysed in 20 mM Tris-HCl, 150 mM NaCl, 0.5% Triton X-100, 0.1% SDS, 1 mM EDTA, and 1 mM sodium orthovanadate. The samples were pre-cleared by centrifugation at 10,000 rpm for 10 min at 4°C, and total protein concentrations were determined using the Bradford protein assay (BioRad, Hercules, CA). For detection of ADAM (a disintegrin and metalloproteinase) proteins, we used lysates from cells that had or had not been transfected with morpholino antisense oligonucleotides. Lysis buffer contained 10 mM 1,10-ortho-phenanthroline to prevent autolysis of the ADAMs. For detection of amphiregulin (AR) shed into cell culture medium, we concentrated the medium 10X using Amicon Centriplus filters with a cutoff of 3 kDa as previously described [4]. For determination of the phosphorylation state of TACE or EGFR, we incubated equal amounts of lysate with anti-TACE or EGFR antibodies and Protein A-agarose beads overnight at 4°C. The lysate-antibody-bead complex was spun down and washed three times with lysis buffer. Following the final wash, 40 µl of SDS gel-loading buffer was added, the mixture was heated at 100°C for 3 min, and proteins were resolved by SDS-PAGE. For immunoblot analysis of the samples listed above, proteins were transferred to nitrocellulose membranes using the Bio-Rad Mini Trans-Blot electrophoretic transfer cell. Membranes were blocked for 1 h at room temperature in phosphate-buffered saline containing 0.1% Tween 20 (PBS/Tween) and supplemented with 5% BSA, then washed with PBS/Tween and incubated with the appropriate antibody overnight at 4°C. After removing primary antibody with several washes of PBS/Tween, the blot was placed in the appropriate horseradish peroxidase-conjugated secondary antibody for 1 hr. After several washes, the antibody-antigen complexes were visualized using the ECL chemiluminescence detection system (GE healthcare Piscataway, NJ).

### Cell-free, TACE catalytic activity assay

NCIH292 cells were grown to semiconfluence in T75 tissue culture flask then starved in serum free medium for 24 h followed by stimulation with SMK for 10 min. The medium was then removed and cells were washed in PBS. Total cell lysates were prepared using CytoBuster™ Protein Extraction Reagent (Cat. No. 71009) in the absence of protease inhibitors. The enzymatic activity of TACE was quantified using the InnoZyme TACE activity kit (Calbiochem), a specific and sensitive assay for measuring human TACE activity in cell lysates and biological samples [49]. The enzymatic activity of TACE in 1 mg protein of SMK or SFM treated cell lysate was measured following the detailed protocol recommended by the manufacturer for assessing activity in biological samples. The effect of phosphatase on cell-free TACE catalytic activity was tested according to the manufacturer recommended protocol modifications for inhibitor screening also [49]. Phosphatase was added to the solution at the indicate concentrations. Fluorescence emission (excitation 320 nm/emission 395 nm) upon cleavage of the quenched fluorogenic peptide (TACE substrate) was monitored with a FLUOstar OPTIMA plate reader. The results are displayed in relative fluorescence units (RFU) and the fluorescence from each sample is corrected by subtracting the fluorescence of the Blank. The mean fluorescence for each sample is calculated from triplicate readings to obtain the final RFU.

### ADAM10 and TACE knockdown by Morpholino Antisense Oligonucleotides

NCIH292 cells were transfected according to the manufacturer's instructions with 2 µM solutions of morpholino antisense oligonucleotides (Gene Tools LLC, Philomath, OR) corresponding to ADAM10, 5′ AATTAACACTCTCAGCAACACCATC- 3′; or ADAM 17, 5′ TCAGGAATAGGAGAGACTGCCT-3′. Thirty h later, cells were lysed for ADAM immunoblot or stimulated with SMK or EGF. ADAMs immunoblots were normalized for loading differences using b-actin. Stimulated cells were used for immunoprecipitation and phospho-EGFR immunoblot as described above.

### Protein kinase C (PKC) activity assay

PKC activity was detected by using PepTag(®) Assay (Promega, WI, USA), a non-radioactive detection method of Protein Kinase C. Cell extracts samples for PKC assay were prepared following the protocol recommended by the manufacturer. The reaction mixture, in a final volume of 25 µl, consisted of 5 µl reaction buffer, PepTag C1 (PKC substrate) 5 µl (0.4 µg/µl), PKC activator solution (DG) 5 µl, peptide protection solution 1 µl and cell extract sample 9 µl. Phosphorylation reaction was allowed to continue for 30 minutes, then 25 µl reaction mixture was subjected to electrophoresis on a 0.8% agarose gel at 100 V for about 20 minutes. After electrophoresis, the PepTag C1 peptides which were phosphorylated and non-phosphorylated were separated, phosphorylated PepTag C1 peptide with negative electricity migrated toward the anode (+), but nonphosphorylated PepTag C1 peptide with positive electricity migrated toward cathode (−), the gel was photographed. Electrophoresis bands on anode represented PKC activity and were analyzed quantitatively.

### PKC isoforms knockdown with siRNAs

H292 cells were cultured in RPMI-1640 medium with 10% FBS, to a 60% density then transfected with PKC isoforms (PKCε, PKCα or PKCδ) siRNAs according to the manufacturer's instructions (Santa Cruz Biotechnology, Santa Cruz, CA). Before the transfection, medium was changed, and new RPMI-1640 medium without FBS and antibiotics was added. The optimal transfection method was the same as previously described [45], [46], [47]. 6 hours after transfection, the medium was changed to new medium with 10% FBS. Thirty hr later, cells were lysed for PKC isoforms immunoblot or stimulated with SMK. PKC isoforms immunoblots were normalized for loading differences using β-actin.

### Effect of SMK treatment on distribution of PKC isoforms

To determine translocation or distribution of PKC, particulate and cytosol fractions were prepared. Cultured H292 cells were treated for 10 min with SFM, SMK or PMA (400nM), upon termination of the experiment, cells were placed on ice, scraped into homogenization buffer (10 mM Tris} HCl, pH 7.4, 250 mM sucrose, 5 mM MgCl2, 50 µg/ml aprotinin, 50 µg/ml leupeptin) (modified from [50], [51]). Cells were homogenized using a Dounce tissue grinder. Cell homogenates were centrifuged at 1,000×g for 10 min at 4°C to discard unbroken cells and nuclei. Supernatants were subjected to additional centrifugation at 10,000×g for 25 min at 4°C. The resulting pellets were resuspended in homogenization buffer and designated as the heavy-membrane fraction. The supernatants were further centrifuged at 150,000×g for 90 min at 4°C and collected as the cytosolic fraction. The protein concentrations in the cytosol (supernatant) and membrane fractions were determined using a Bradford assay. Samples were concentrated using the deoxycholate/trichloroacetic acid precipitation method and reconstituted in Laemmli sample buffer to yield 3 mg/ml protein. A 200 µg aliquot of total protein (cytosolic or membrane fractions) was subjected to SDS/PAGE; proteins were transferred to a PVDF membrane, incubated with anti-PKC isoforms antibodies and developed as described above.

### Enzyme-linked immunosorbent assay (ELISA) for Amphiregulin Shedding

H292 cells were seeded into T75 flask and incubated for 18 h. Upon serum deprivation for 24 h, the medium was then removed and cells were washed intensively in PBS and culture medium followed by stimulation with fresh serum-free medium (SFM) or SMK-containing medium for 10 min. The cell medium was then collected for analysis. As a control, cell medium was collected immediately after it was added (t = 0). In some experiments cells were subjected to pre-incubation with TACE/MMP inhibitor (GM6001, 20 µg/ml), PKC inhibitor (BIS), SRC kinase inhibitor (PP2) or ROS inhibitor (DMTU, µm) or the appropriate vehicle control 2 hours prior to stimulation with SMK-containing medium. The release of amphiregulin (AR) into the culture medium was measured by sandwich ELISA (R&D Systems) using monoclonal anti-AR capture antibody (MAB262) and biotinylated polyclonal detection antibody (BAF262). Plate preparation and assay procedure were performed according to the manufacturer's recommendations using tetramethyl benzidine as a substrate. The absorbance at 455 nm was read with a reference wavelength of 650 nm using an ELISA plate reader. AR concentrations for each sample were calculated after generating a standard curve using the dilution series of human recombinant AR protein.

### Detection of Reactive Oxygen Species (ROS) Production

Mouse embryonic fibroblasts (MEFs) null for the three SRC family members SRC, Yes and Fyn, (SYF cells) were grown on coverslips were incubated for 30 min prior to SMK or SFM exposure with 10 µM 2,7-Dichlorodihydrofluorescein diacetate, H2DCFDA (Molecular Probes, Eugene, OR) as previously described [4]. Fluorescent images were captured using a Nikon Eclipse E600 microscope equipped with epifluorescence optics and a Zeiss Axiocam digital camera.

### SRC kinase activation

NCIH292 cells were grown to semiconfluence in 35 mm culture dishes then starved in serum free medium for 24 h then cells were preincubated with PP2 (10 µM), BIS or DMTU for 2 h prior followed by stimulation with SMK for 10 min. The medium was then removed and cells were washed in PBS. Total cell lysates were prepared as described above. The lysates were subjected to 12% polyacrylamide gel electrophoresis (SDS-PAGE), transferred to a PVDF membrane and blotted with specific antibody for phosphorylated SRC at Tyr-416. Blots were then stripped and reprobed using specific antibodies directed total SRC.

### Cell growth assays

Cell growth was determined by Cell Titer Blue cell viability assay (Promega, Madison, WI). Briefly, 3000 cells of H292 control or H292 Knockdown for TACE, PKCε or PKCδ, were seeded per well of a 96 well plate. Cultures were maintained in a 37°C incubator in a humidified atmosphere of 5% CO2. Medium was removed and replaced with fresh medium every 2 days. PKC inhibitor BISindolylmaleimide (BIS) was added on day 0, 2 Hr prior to a 2 Hr treatment with serum-free medium (SFM) or smoke-containing medium and replenished at the time of medium change every 2 days. After 48 Hr of incubation at 37°C, counts of viable cells were determined with the Cell Titer Blue assay according to the manufacturer's instructions.

### Culture of HBE Cells

Primary human airway epithelial cells (HBE) were obtained from Clonetics (San Diego, CA) and were cultured under conditions recommended by the vendor. Primary HBE cells were plated 1×10^5^ cells/cm^2^ onto Transwell polycarbonate membranes, 0.4-µm pore diameter; Corning, Inc., NY, precoated with human placental collagen (15 mg/cm2; Sigma-Aldrich, St. Louis, MO). Cells were grown in defined ALI medium at an air liquid interface producing highly differentiated and functional replicas of the airway epithelium for approximately 15 days [52], [53]. As described above for NCIH292 cells, following a 10 min treatment of HBE cells with serum-free medium (SFM) or smoke-containing medium (SMK), cell extract were assessed for TACE and EGFR phosphorylation, PKC activity and co-localization of TACE with PKCε. Also, cell culture medium was collected from the latter cells and assayed for amphiregulin release as described above.

### Statistical analysis

Student's t-test was used to compare data between two groups. Values are expressed as mean 6 SD of at least triplicate samples. P<0.05 was considered statistically significant.

## Supporting Information

Figure S1 **Smoke-induced changes of PKC activity and TACE activity in NCIH292 are not affected by a 2-hr incubation with vehicle (0.1% DMSO) used for inhibitors (DMTU, PP2 and BIS) studies.** (A) NCIH292 cells were incubated or not with vehicle control (0.1% DMSO) for 2 h prior to stimulation with smoke (SMK) for 10 min. Cell extracts samples were assessed for TACE phosphorylation, EGFR phosphorylation and PKC activity as described in Figs. 2 and 3. (B) NCIH292 cells were incubated with 5 µM of the general PKC inhibitor, bisindolylmaleimide (BIS) or vehicle control (0.1% DMSO) for 2 h prior to stimulation with smoke (SMK) for 10 min. Total cell lysates were prepared and TACE activity was measured as described in Fig. 1. (C) Cell culture medium was also collected from the latter cells and assayed for amphiregulin release by ELISA assay as described in Fig. 2. Double asterisks indicate significantly different from SFM (p<0.01).
(TIFF)

Figure S2 **Exogenous Reactive Oxygen Species (ROS) stimulate PKC activity in SRC dependent manner.** NCIH292 cells were incubated for 2 h with SRC kinase inhibitor (PP2) (10 µM) or vehicle control (0.1% DMSO), prior to stimulation with SFM, or H2O2-containing SFM (H2O2) for 10 min. Cell extracts samples were assessed for PKC activity as described in Fig. 2.
(TIFF)
