# Supplementary figures and images for: TACE/ADAM-17 Phosphorylation by PKC-Epsilon Mediates Premalignant Changes in Tobacco Smoke-Exposed Lung Cells
Source: PLoS One. 2011 Mar 15;6(3):e17489. doi: 10.1371/journal.pone.0017489 (PMC3057966; doi:10.1371/journal.pone.0017489)

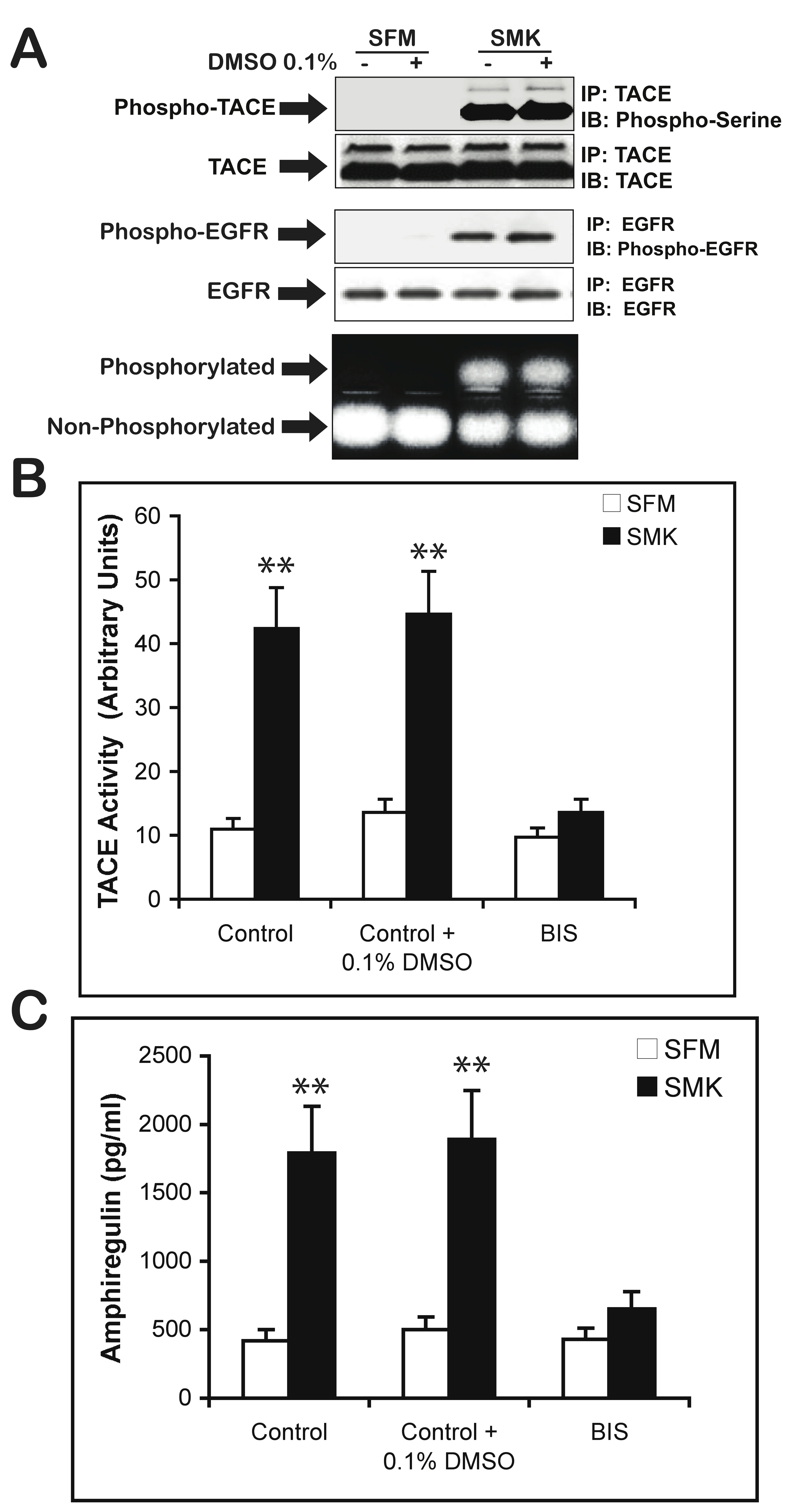

Supplement: Figure S1 — Smoke-induced changes of PKC activity and TACE activity in NCIH292 are not affected by a 2-hr incubation with vehicle (0.1% DMSO) used for inhibitors (DMTU, PP2 and BIS) studies. (A) NCIH292 cells were incubated or not with vehicle control (0.1% DMSO) for 2 h prior to stimulation with smoke (SMK) for 10 min. Cell extracts samples were assessed for TACE phosphorylation, EGFR phosphorylation and PKC activity as described in Figs. 2 and 3. (B) NCIH292 cells were incubated with 5 µM of the general PKC inhibitor, bisindolylmaleimide (BIS) or vehicle control (0.1% DMSO) for 2 h prior to stimulation with smoke (SMK) for 10 min. Total cell lysates were prepared and TACE activity was measured as described in Fig. 1. (C) Cell culture medium was also collected from the latter cells and assayed for amphiregulin release by ELISA assay as described in Fig. 2. Double asterisks indicate significantly different from SFM (p<0.01). (TIFF) [file pone.0017489.s001.tiff]

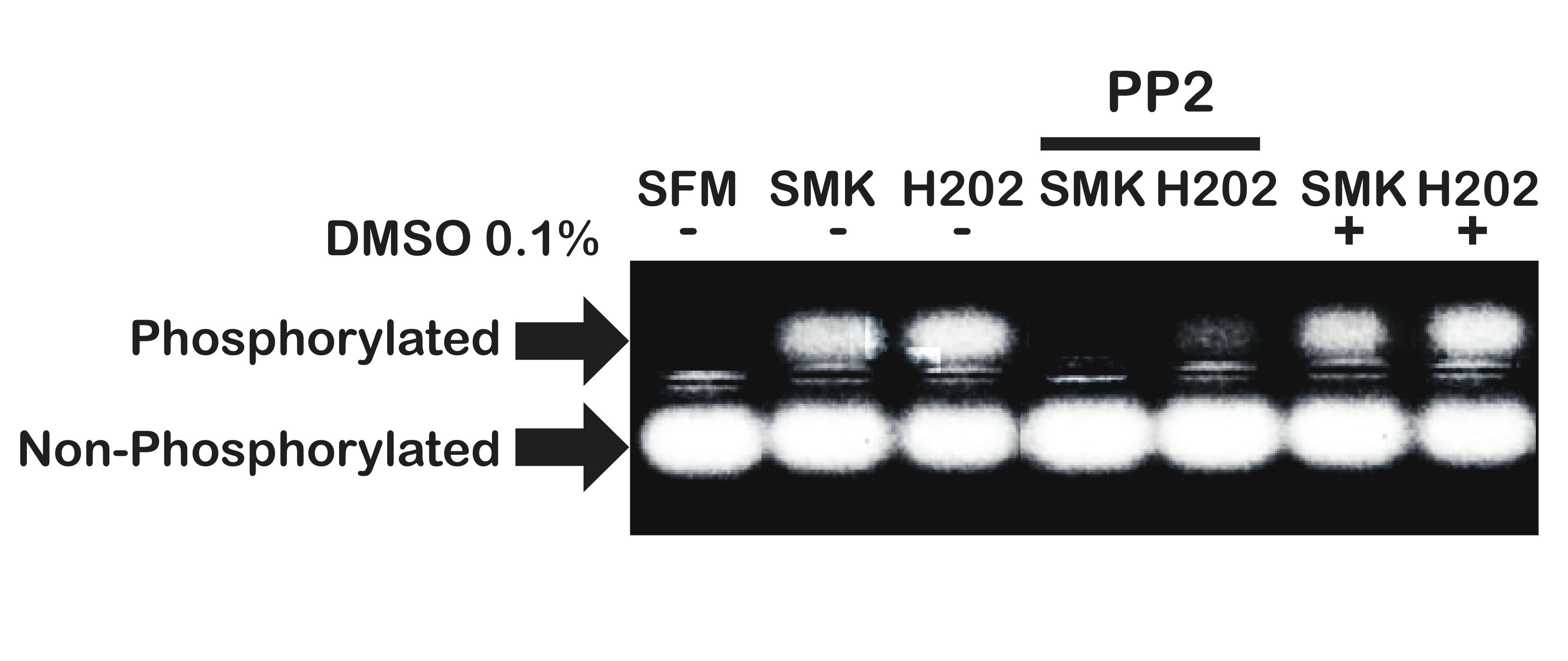

Supplement: Figure S2 — Exogenous Reactive Oxygen Species (ROS) stimulate PKC activity in SRC dependent manner. NCIH292 cells were incubated for 2 h with SRC kinase inhibitor (PP2) (10 µM) or vehicle control (0.1% DMSO), prior to stimulation with SFM, or H2O2-containing SFM (H2O2) for 10 min. Cell extracts samples were assessed for PKC activity as described in Fig. 2. (TIFF) [file pone.0017489.s002.tiff]
